# Retained Functionality of Atherosclerotic Human Arteries Following Photoactivated Linking of the Extracellular Matrix by Natural Vascular Scaffolding Treatment

**DOI:** 10.1007/s12265-020-10063-y

**Published:** 2020-08-03

**Authors:** Ejaz Ansari, Blake Anderson, Katalin Kauser

**Affiliations:** 1 REPROCELL Europe Ltd, Glasgow, UK; 2 Alucent Biomedical Inc., 675 Arapeen Dr Ste 102, Salt Lake City, UT 84108 USA

**Keywords:** Inflammation, Natural vascular scaffolding, Peripheral arterial disease, Vessel reactivity

## Abstract

In this study, we investigated natural vascular scaffolding (NVS) treatment on vascular functionality using freshly isolated human popliteal arteries in vitro. Arteries were exposed to intraluminal NVS treatment consisting of a compound (4 amino-1,8-naphthalimide) photoactivated by a 450-nm light-emitting light fiber placed inside the artery. This procedure results in covalent linking between the extracellular matrix proteins to achieve a larger vessel diameter post-angioplasty and minimizing elastic recoil. Immediately following NVS treatment, rings were cut from the treated arteries and mounted in organ baths for contractility testing in response to U46619 and sodium nitroprusside. We also investigated the effect of NVS treatment on IL-6 cytokine release from vascular rings following a 4-h organoculture post-NVS treatment. Based on our results, we conclude that exposure of the vessels to NVS treatment does not adversely affect the contractile responsiveness of the vascular smooth muscle and exerts no pro-inflammatory effect.

**Figure:**
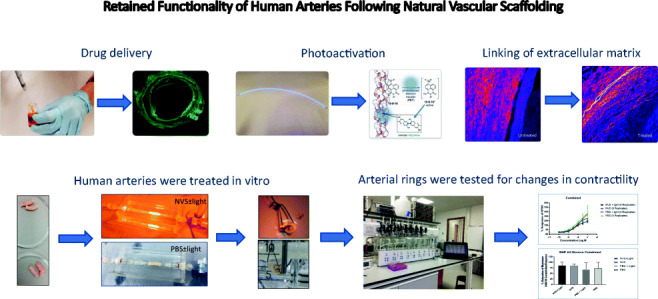
Graphical abstract

Graphical abstract

## Introduction

According to recent estimates, approximately 8.5 million Americans over the age of 40 years and more than 200 million individuals worldwide have various forms of peripheral arterial disease (PAD), affecting the lower extremities [1, 2]. In addition to the progressive decline in ambulatory function and poor quality of life, individuals with PAD are also at increased risk of cardiovascular mortality and morbidity [3]. Currently, PAD is one of the costliest medical indications and, with the aging of the population, it will likely become a major economic and healthcare burden [4].

Over the past decades, PAD has been mainly treated with minimally invasive balloon angioplasty and stenting. Revascularization of the ischemic limb is the cornerstone of PAD therapy as, without it, many patients will undergo a major limb amputation within 1 year of diagnosis [5]. Although more effective than medical therapy, endovascular revascularization is nevertheless associated with significant complications, namely restenosis and the need for repeat revascularization procedures [6, 7]. Stenting of coronary arteries has shown promising results in terms of long-term vessel patency, and the use of contemporary stents has significantly reduced the incidence of coronary restenosis [8, 9]. However, because the arteries of the lower extremities are in a unique dynamic environment and undergo extensive deformation, twisting, and compression with each limb flexion, using stent implants in the peripheral arteries often leads to vessel dissections [10, 11], stent malapposition [12], and fractures [13], all associated with restenosis and reocclusion and, therefore, lower patency. Therefore, these procedures are associated with restenosis which, in the femoropopliteal arteries, can reach rates as high as 40% [6, 7, 14, 15]. Consequently, the search for novel treatment approaches offering an alternative to stenting has gained interest.

Currently in use and in development, angioplasty balloons coated with antiproliferative agents target the proliferating cellular components of the vessel wall to reduce reocclusion due to hyperplasia and inflammation elicited by the interventional treatments [16–18]. Paclitaxel has emerged as the most potent anti-restenotic agent for the peripheral arteries [19], but the recent observation by Katsanos and colleagues of higher rates of major amputations and increased mortality has raised concerns as to its safety [20]. Although very effective at reducing restenosis, due to the rigid nature of many stents coated with cytotoxic drugs, they also are detrimental to the functional reactivity of the vessel wall ultimately disrupting the physiological healing processes allowed by the restoration of blood flow. Accordingly, research efforts have focused on the development of innovative therapies addressing the limitations of endovascular procedures to improve the outcome and, therefore, the quality of life of patients with PAD. A promising alternative to rigid stenting for peripheral procedures is the targeting of native structural proteins, such as collagen and elastin, present in the extracellular matrix (ECM) of the vessel wall following balloon dilatation. Used as an adjunct to balloon angioplasty, natural vascular scaffolding (NVS®, Alucent Biomedical, Salt Lake City, UT) delivers a photoactivatable drug to the site of the lesion which, upon activation by a 450-nm wavelength light from an intraluminally placed light fiber, creates covalent bonds between amino acid components of the ECM resulting in a natural, flexible scaffold maintaining the lumen size, while preserving the natural biomechanical behavior of the arterial wall [21, 22]. This technology is currently being evaluated in clinical development with the potential to offer an alternative to stenting.

The aim of the present study was to investigate if NVS treatment affects smooth muscle contractility and inflammatory responses of peripheral arteries using freshly isolated human popliteal arterial segments, in vitro.

## Methods

### Materials

Photoactivatable small molecule (4 amino-1,8-naphthalimide or 10-8-10), NVS solution (2 mg/mL 10-8-10 dissolved in phosphate-buffered saline (PBS)), light fiber, and light source were supplied by Alucent Biomedical. The thromboxane A_2_ mimetic U46619 was obtained from R&D Systems (Oxfordshire, UK), and sodium nitroprusside (SNP) and acetylcholine were obtained from Sigma-Aldrich (Dorset, UK). For U46619, a 1-mM stock solution was prepared in DMSO while SNP (100 μM) and acetylcholine (10 μM) were prepared in deionized water. All stock solutions were stored at − 20 °C until use. The cell culture media (Gibco) for the organoculture experiments was obtained from Thermo Fisher (UK). The 96-well plates were sourced from Costar, Corning Incorporated (UK).

All organ bath and culture experiments were conducted at REPROCELL Europe Ltd. (Glasgow, UK) while the fluorescent and multiphoton imaging were conducted at Alucent Biomedical.

### Tissue Preparation and Natural Vascular Scaffolding Treatment

Fresh human popliteal arteries were obtained at autopsy (5 donors, age range of 55 to 82 years) as per REPROCELL’s human tissue protocol TPO-059-UK. All donors had serious heart and other health conditions including coronary artery disease, congestive heart failure, myocardial infarction, chronic obstructive pulmonary disease, cardiac stents, type 2 diabetes mellitus, and end-stage kidney disease. All excised arteries were stored in Dulbecco’s modified eagle medium (DMEM; Thermo Fisher, UK) on wet ice until use.

Immediately upon arrival to the laboratory (cold ischemic time of 26 to 46 h), the popliteal arteries were placed in a cold physiological saline solution containing 119.0 mM NaCl, 4.7 mM KCl, 1.2 mM MgSO_4_, 24.9 mM NaHCO_3_, 1.2 mM KH_2_PO_4_, 2.5 mM CaCl_2_, and 11.1 mM glucose, dissected free from surrounding tissue and cut into segments of approximately 3–4 cm in length. The experimental methods have been described in detail previously [21]. Briefly, the popliteal segments were attached to a cannula on both ends of a perfusion organ chamber allowing for the intraluminal drug delivery. The cannulated segments were randomly allocated to 1 of 4 experimental groups: (1) 5-min incubation with 2 mg/mL NVS solution (*n* = 5), (2) 5-min incubation with 2 mg/mL NVS solution and a 1-min 450 nm intraluminal light activation (*n* = 5), (3) 5-min incubation with the vehicle (PBS) (*n* = 5), and (4) 5-min incubation with the vehicle (PBS) and a 1-min 450-nm intraluminal light activation (*n* = 4). Following treatment, and for each experimental group, the segments were cut into four 2–3-mm rings with, one ring per group processed immediately for histology studies, and fluorescent and multiphoton imaging to investigate drug penetration and confirm the ECM density change due to photoactivated linking of collagen and elastin following NVS treatment, one ring per group for the organoculture experiments, and the last two rings per group for the organ bath experiments.

### Histology Methods

Each artery ring was snap-frozen in liquid nitrogen (LN2, Technifab), cryo-sectioned in either 5- or 10-μm-thick sections using a Leica CM1850 cryostat and adhered to charged glass slides without coverslips. The 5-μm-thick sections were stained with hematoxylin and eosin and Masson’s trichrome for descriptive histological and morphometric analysis. The histochemical staining was performed using reagents and staining protocols from Newcomer Supply (Middleton, WI) with appropriate controls. Imaging of the stained segments was performed on a Zeiss Axio Scan.Z1 bright-field setting. The 10-μm-thick sections were used for the determination of the 10-8-10 depth of penetration into the arterial wall using Zeiss Axio Scan.Z1 and filters for excitation wavelength 450–490 nm. The depth of 10-8-10 penetration was quantitated by comparing the autofluorescence of the PBS-treated rings to that of rings from NVS-treated arteries using the profile feature in the ZEN 2.5 lite software (Zeiss, Germany).

For second harmonics generation (SHG) imaging, the 10-μm-thick slides were placed in a petri dish and covered with PBS at least 1–2 cm above the slide. The SHG images were performed on a Bruker Prairie multiphoton confocal microscope with a Ti-sapphire tunable laser. The microscope settings were as follows: × 25 Nikon objective, laser wavelength set to 800 nm at a power setting of 150 with a pixel dwell time of 20.8 ms, and backward PMT detection method with a SHG cube bypass filter at 377/50. All images were collected at constant power and wavelength. Minor setting adjustments were required as to not oversaturate the detector. The FIJI/ImageJ was used to calculate the intensity values of the artery sections.

### Organ Bath Experiments

Two rings from each group were mounted in 25-mL organ baths (Panlab SI, Barcelona, Spain) containing physiological saline solution, oxygenated with a gas mixture of 95% O_2_ and 5% CO_2_ and maintained at a temperature of 37 °C for the isometric tension recording of vascular contractility. Changes in tension were detected using an isometric transducer (TRI202PAD, Panlab SI, Barcelona, Spain) and the output signals were processed using the PowerLab16/35 data acquisition software (ADInstruments). Recordings of the outputs were made on the LabChart software (version 7.3.8, ADInstruments).

The artery rings were allowed to equilibrate for at least 30 min, stretched to a standard tension of 1.0 g (± 0.1 g), and allowed to reach a steady tension. Each ring was then exposed to a high potassium saline solution (KPSS) (62.5 mM) with sequential washes in between. Following stretching and reactivity testing by a high potassium saline solution, the rings were subjected to increasing concentrations of the thromboxane analog, U46619 (100 pM to 100 nM) to elicit a contractile response. For each concentration, the rings were exposed for a minimum of 5 min or until the response reached a plateau before adding the next concentration. At the end of the U46619 curve, acetylcholine (10 μM) was added to each bath followed by high concentration of sodium nitroprusside (SNP, 100 μM) to induce maximum relaxation and the responses were allowed to reach a plateau. The maximum constriction or relaxation value at each concentration was analyzed and the values were converted to a percentage of maximum high potassium saline solution responses. Popliteal vascular rings from *n* = 5 donors were used in the contractility studies; 10 replicate rings/donors = 2 rings of each donor segment were studies under each conditions, unless rings did not contract in response to the high potassium saline solution (62.5 mM) at the start of the testing.

### Organoculture Experiments

One artery ring from each experimental group was cultured for 4 h at 37 °C in DMEM culture medium at an atmosphere of humidified air with 5% CO_2._ The supernatants were collected and stored at − 80 °C before being analyzed for interleukin-6 (IL-6) by multiplex ELISA on Luminex Magpix system using Luminex xMAP compatible bead technology (Luminexcorp). Each analyte was quantified by interpolation against a standard curve generated on the same 96-well analysis plate.

### Statistical Analysis

A one-way ANOVA (analysis of variance) was used for all bar graphs (Figs. 2, 4, and 5) and a two-way ANOVA with Dunnett’s ad hoc test was used for the organ bath cumulative dose-response curves (Fig. 3). A *P* value ˂ 0.05 was considered statistically significant. All analyses were performed with the GraphPad Prism software (version 8.2, GraphPad Software Inc., San Diego, CA).

## Results

### Histology Studies

**Fig. 1:**
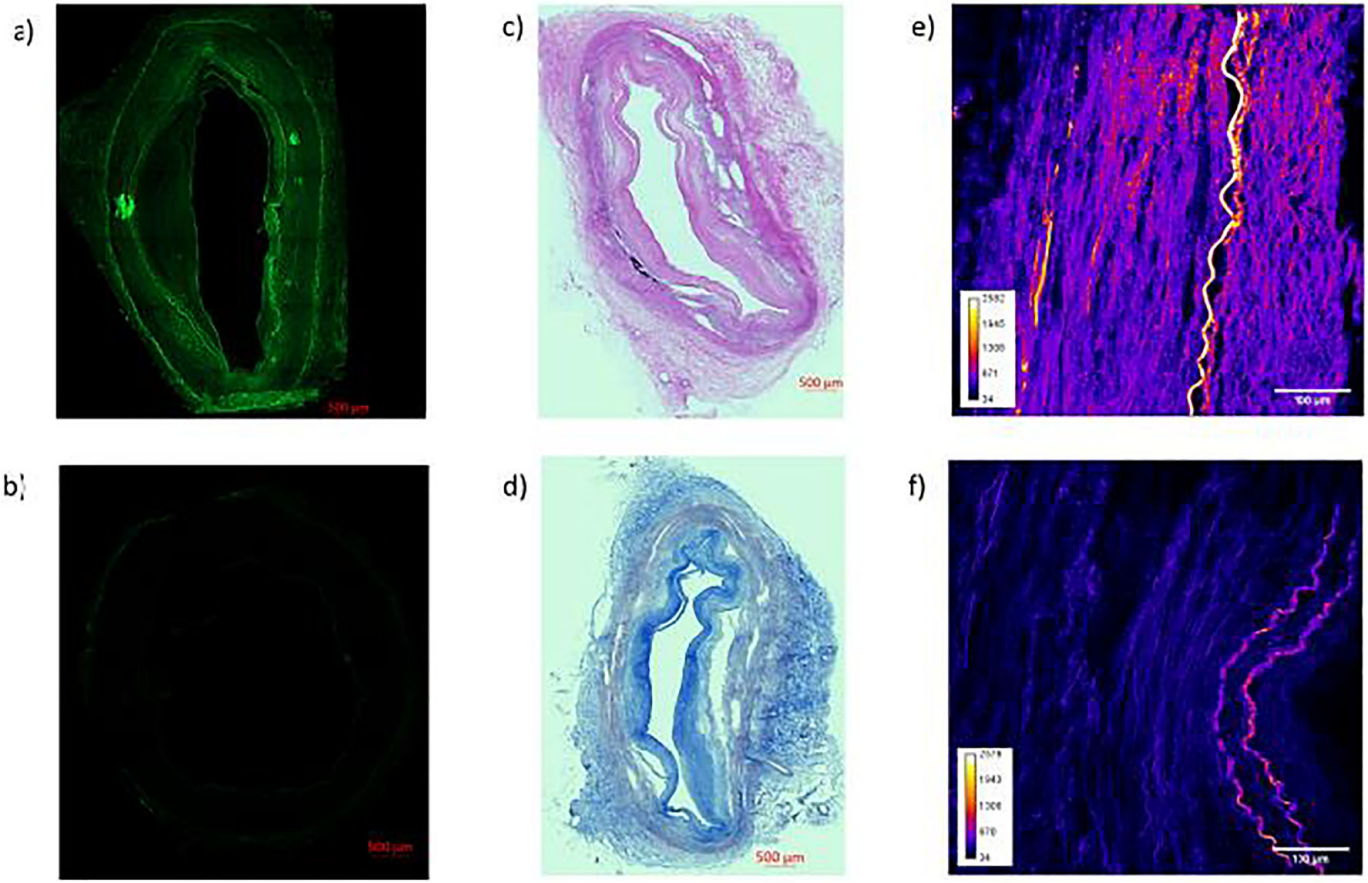
Representative fluorescent imaging demonstrating the penetration of the light activatable compound across the arterial wall (**a**) compared with the autofluorescence of the control tissue (**b**), hematoxylin and eosin (**c**), and Masson’s trichrome (**d**) staining revealing the presence of significant atherosclerosis in the treated rings and (**e**) representative multiphoton imaging revealing a denser medial fiber network in NVS + light-treated arteries compared with controls (**f**)

Cross-sections of each arterial ring were prepared for histological examination following experimental treatment to verify the presence of atherosclerotic disease, to elucidate drug distribution and confirm the photoactivation-induced changes in the ECM following NSV treatment. As shown in Fig. 1, the hematoxylin and eosin (panel c) and the Masson’s Trichrome (panel d) staining revealed the presence of significant atherosclerotic disease in each treated vascular rings. Based on fluorescent imaging and compared with the autofluorescence of a ring treated with PBS + light (panel b), light activatable 10-8-10 was homogenously distributed across the arterial wall (panel a). Finally, multiphoton imaging revealed a denser medial fiber network in NVS + light-treated arteries (panel e) compared with rings treated with PBS + light (panel f).

### Organ Bath Studies

**Fig. 2:**
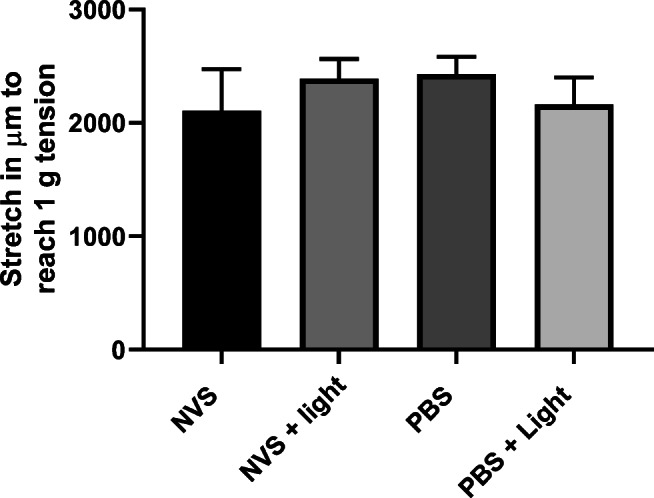
Stretch required (in μm) to reach 1 g (± 0.1 g) tension in isolated human popliteal artery rings pre-treated with NVS, NVS + light, PBS, or PBS + light. All groups showed similar stretch properties

**Fig. 3:**
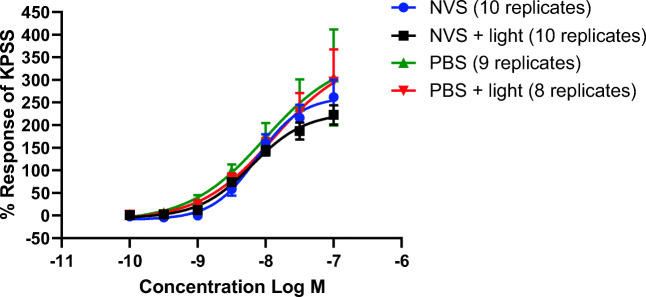
Cumulative dose-response curves to U46619 over the concentration range of 100 pM to 100 nM in human isolated popliteal artery rings pre-treated with NVS, NVS + light, PBS, or PBS + light. Results are expressed as a percentage of the maximal high potassium saline solution response. Non-linear regression of each data set is displayed. Two-way ANOVA with Dunnett’s ad hoc test showed no significant differences between the groups

As measured by the level of stretch required by each artery ring to reach a tension of 1 g (Fig. 2), pre-treatment with NVS ± light did not alter the distensibility compared with rings pre-treated with PBS ± light (*P* = NS). Expressed as a percentage of the maximal KPSS response (mean values are NVS = 1.88; NVS + light = 1.28; PBS = 1.21; PBS + light = 1.29), with all rings showing similar contraction responses over all U46619 concentrations with similar pEC50 and *E*_max_ values Table 1). The cumulative dose-response curves of U46619 are presented in Fig. 3.

**Table 1:** pEC50 and *E*_max_ values of U46619 for each group

	NVS	NVS + light	PBS	PBS + light
pEC_50_	8.13	8.20	8.02	7.89
*E*_max_	263.1	228.1	351.8	353.1

**Fig. 4:**
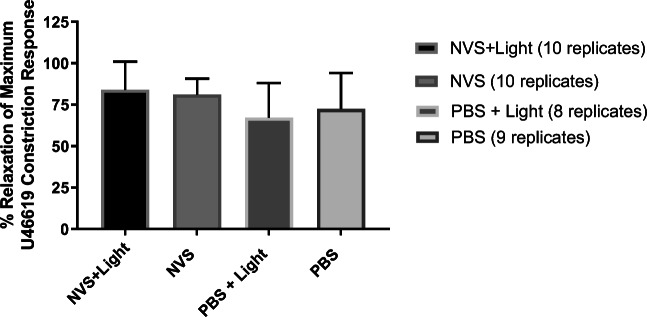
Effect of high concentration of sodium nitroprusside (SNP 100 μM) in human isolated popliteal artery rings pre-treated with NVS, NVS + light, PBS, or PBS + light. All rings were challenged with U46619 (100 nM) prior to the administration of sodium nitroprusside. Results are expressed as a percentage relaxation of the maximal U46619 constriction response

Immediately after U46619 treatment, the rings were challenged with acetylcholine (10 μM) and SNP (100 μM) to determine if NVS treatment altered their relaxation properties. As shown in Fig. 4, similar relaxation responses to SNP were observed in all the groups (*P* = NS), indicating that treatment with NVS ± light did not affect smooth muscle relaxation. However, when the rings were challenged with acetylcholine, no relaxation was observed in any rings (data not shown), likely indicating damaged endothelium due to atherosclerosis despite careful handling of the tissues.

### Organoculture Studies

**Fig. 5:**
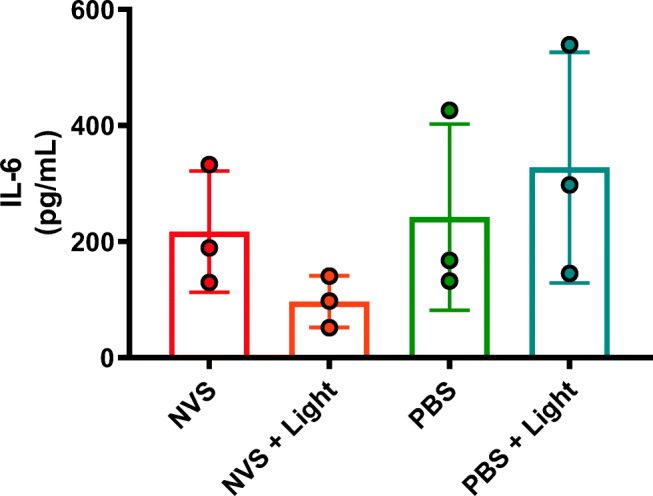
IL-6 levels in supernatants collected from cultured human isolated popliteal artery rings (*n* = 3) pre-treated with NVS, NVS + light, PBS, or PBS + light

To determine if NVS treatment was associated with increased inflammation, we measured the level of IL-6 from the supernatants collected from the cultured popliteal artery rings (Fig. 5). The levels of IL-6 were found to be similar between all groups (*P* = NS) albeit slightly lower in the NVS ± light groups than the rings treated with PBS ± light, suggesting that NVS treatment does not increase inflammation and may even exert an anti-inflammatory effect.

## Discussion

Natural vascular scaffolding uses a small molecule (substituted 1,8-naphthalimide) which, upon light activation, catalyzes the linkage between collagen and elastin present in the ECM thereby stabilizing these structural proteins and creating a scaffolding effect without altering the natural biomechanics of the treated vessel [21, 22]. Due to the linkage between elastin and collagen, the elastin fibers become elongated, preventing the elastic recoil usually observed shortly after percutaneous transluminal angioplasty [23].

The present study was conducted to characterize the smooth muscle contractile properties of freshly isolated human popliteal arteries under NVS treatment. The main findings are as follows: (1) the drug distribution of the light activatable compound was homogeneous across the artery wall, (2) treated arteries presented a denser medial fiber network, (3) NVS did not alter the vascular smooth muscle contractile and dilatory properties of the arteries, and (4) no pro-inflammatory response was associated with the use of NVS. Overall, our results indicate that NVS treatment allows normal functional contractility in human popliteal arteries without altering vascular inflammation of the atherosclerotic vessel wall. These findings support the possibility of NVS treatment to become an attractive alternative to current endovascular devices for the treatment of lower extremity PAD.

Results from experimental studies using porcine carotid arteries showed that the luminal gain following balloon angioplasty and NVS treatment was significantly greater with NVS than angioplasty alone [21, 22], a luminal gain not associated with stiffening of the arterial wall. These results also confirmed that NVS was only effective if activated by light. Exposure to light alone did not affect the luminal gain [22]. Results of our study confirmed that NVS treatment did not alter the distensibility of human popliteal arteries, as it was of the same magnitude as that of the control arteries treated with PBS. Although the mechanical properties between arteries from different vascular beds may show some inherent differences such as the muscular femoropopliteal artery and the elastic carotid artery, due to their respective arterial wall composition, the NVS treatment did not adversely affect the vascular properties of the human popliteal arteries. In addition, NVS treatment also preserved smooth muscle functionality as the arteries retained both their contractile properties when challenged with increasing concentrations of the thromboxane A_2_ analog, U46619 and their vasodilatory properties when challenged with SNP. However, neither the NVS-treated nor any of the control arteries responded to acetylcholine stimulation. This is likely due to the presence of atherosclerosis and resulting endothelial dysfunction.

Besides evaluating the effects of NVS on vascular reactivity, we also determined if the treatment was associated with an increased inflammatory response. IL-6 release from isolated atherosclerotic vascular segments over a 4-h incubation under organ culture conditions has been used as a reliable indicator of the presence of vascular inflammation due to atherosclerosis or to measure changes in inflammation as a response to treatments [24, 25]. IL-6 has also recently shown a strong and independent association with atherosclerotic cardiovascular disease events in a multi-ethnic study of atherosclerosis including over 6000 participants [26]. IL-6 measurement improved the prediction of incident heart failure, stroke, and all-cause mortality, particularly among statin users [26]. Based on the IL-6 immunoassay, NVS did not increase the secretion of IL-6 as the levels were similar to those of the PBS-treated arteries. Interestingly, the IL-6 levels were numerically lower in the NVS treatment samples, suggesting a potential anti-inflammatory effect by the treatment. However, because of the small sample size, it is unclear if this is due to a true anti-inflammatory effect or the result of the variability of the existing inflammation in the vessel wall inherent to the underlying atherosclerotic disease, as evidenced also by the lack of endothelium-dependent relation to acetylcholine.

The main limitations of the study are that (1) our results were derived from in vitro experiments which cannot be directly extrapolated to humans; (2) because of the acute nature of our model, it is unknown if these effects will translate into providing a long-term benefit by the NVS treatment; and (3) we only evaluated NVS-mediated inflammation through the release of the cytokine IL-6. Nevertheless, as diseased human popliteal arteries were used instead of animal arteries, we believe our observations provide significant insights into the translational safety of NVS treatment as we approach clinical studies in development.

In conclusion, NVS treatment shows promise in terms of retained vessel functionality without increasing the inflammatory response or stiffening of the vessels. Our results therefore suggest a clear differentiation of NVS treatment compared with stenting with or without cytotoxic drugs to support maintained vascular patency as standard endovascular procedures. The durability of the scaffolding effect has been investigated in chronic animal studies and clinical testing of the technology has recently started.

## Abbreviations

ECM: Extracellular matrix
IL-6: Interleukin-6
NVS: Natural vascular scaffolding
PAD: Peripheral arterial disease
PBS: Phosphate-buffered saline
